# Orthogonal Experimental Study on Mix Proportion Optimization and Mechanical Properties Comparison of Lightweight Aggregate Concrete Made with Recycled Glass Pumice and Ceramsite

**DOI:** 10.3390/ma19132871

**Published:** 2026-07-05

**Authors:** Xiao Li, Ruirui Qian, Zhihao Zhai, Chengquan Wang, Mingyu Fang, Xinquan Wang, Yuxuan Ding, Tengfang Dong

**Affiliations:** 1 School of Engineering, Hangzhou City University, Hangzhou 310015, China; lix@hzcu.edu.cn (X.L.); 15906672982@163.com (R.Q.); wangxq@hzcu.edu.cn (X.W.); 2 College of Civil Engineering and Architecture, Guilin University of Technology, Guilin 541004, China; 18306162177@163.com (Z.Z.); dingyuxuan0226@163.com (Y.D.); 3 CCTEG Chongqing Engineering (Group) Co., Ltd., Chongqing 400042, China; yu373344569@163.com; 4 College of Civil Engineering and Architecture, Zhejiang University, Hangzhou 310058, China; 12412095@zju.edu.cn

**Keywords:** recycled glass pumice, lightweight aggregate concrete, orthogonal experimental design, compressive strength, ceramsite, mix design

## Abstract

To explore the feasibility of using recycled glass pumice (microcellular glass pumice aggregate, MGPA) as a substitute for traditional lightweight aggregates and to compare its mechanical performance with that of expanded clay ceramsite, this study systematically investigated the effects of water–cement ratio (0.40–0.46), cement content (330–360 kg/m^3^), fine MGPA replacement ratio (0–100%), and coarse MGPA replacement ratio (0–100%) on the dry density and compressive strength of lightweight aggregate concrete through an orthogonal experimental design. The results show that the bulk density of coarse MGPA (312 kg/m^3^) is only 46% of that of ceramsite (678 kg/m^3^), while its cylinder compressive strength (3.36 MPa) is slightly lower. The range analysis indicates that the dry density of MGPA concrete is primarily influenced by the replacement ratio of coarse aggregate, followed by fine aggregate replacement, water–cement ratio and cement content; the lowest dry density (1445 kg/m^3^) was obtained with a water–cement ratio of 0.46, cement content of 330 kg/m^3^, 100% replacement of coarse MGPA, and partial replacement of fine MGPA (mixture S19). For the 28-day compressive strength, the influencing factors rank as coarse aggregate replacement > water–cement ratio ≈ cement content > fine aggregate replacement. In comparison with the ceramsite concrete reference under the respective mix designs tested in this study, the optimal MGPA concrete exhibited only 4.6% higher dry density but achieved a significantly higher compressive strength of 40.0 MPa, compared with 20.5 MPa for the ceramsite mixture. The specific strength (strength/density ratio) of MGPA concrete is about 1.87 times that of ceramsite concrete. Both types of lightweight aggregate concrete reached 77–80% of their 28-day strength at 7 days. Overall, recycled glass pumice is a promising alternative to ceramsite for lightweight concrete, especially when both high strength and low weight are required for precast components, provided that its long-term durability (particularly ASR resistance) is verified in future studies.

## 1. Introduction

Lightweight aggregate concrete (LWAC) has become an important research direction in civil engineering because of its ability to significantly reduce structural dead load, seismic response, and building energy consumption [1,2]. With the growing global emphasis on building energy conservation and sustainable development, the use of waste materials to produce novel environmental-friendly lightweight aggregates has attracted increasing attention. Approximately 7% of the global solid waste is waste glass, but most of it is landfilled, causing serious environmental problems and natural resource depletion [3]. Although waste glass can be infinitely recycled without loss of quality, the high cost of separation and cleaning limits its recycling rate. Therefore, large-scale utilization of waste glass in building materials is both environmentally and economically beneficial [4].

Foamed glass aggregate, a porous lightweight inorganic material produced by sintering waste glass powder with foaming agents, has been developed as a promising green building material [5]. Osfouri et al. [6] prepared sustainable structural lightweight concrete by replacing natural coarse aggregate with foam glass aggregate at volume replacement ratios of 20%, 40% and 60%, and found that even at 100% replacement the 28-day compressive strength reached 23.3–27.9 MPa, meeting the requirements of ASTM C330. Bian et al. [7] developed a self-foaming cold-bonded process to produce lightweight aggregates from waste glass powder and incineration bottom ash; increasing the glass powder content effectively increased the pore size and reduced the aggregate density. Mydin et al. [8] replaced natural sand with crushed soda-lime glass waste at mass ratios of 5–50% and reported that the optimal replacement was 20%, at which the compressive, flexural and splitting tensile strengths of the foamed concrete increased by 17.7%, 39.4% and 43.8%, respectively; SEM analysis showed that 20% replacement significantly reduced the pore diameter and improved the uniformity of the pore structure. Cao et al. [9] incorporated glass microspheres into foamed concrete and demonstrated that they significantly reduced density while maintaining good mechanical performance. Furthermore, review studies on the application of waste glass in cementitious composites have indicated that replacing 20–30% of natural aggregate with waste glass can substantially improve concrete strength, but it also brings the risk of expansion and cracking due to alkali–silica reaction (ASR), which has become a major durability issue limiting the widespread use of glass aggregates [10,11].

Hollow glass microspheres (HGMs) have also received wide attention for use in ultra-lightweight cementitious composites. Lee et al. [12] investigated the shear behavior of high-strength lightweight composites containing HGMs and carbon nanotubes; the material achieved a compressive strength of 87.8 MPa and a dry density of only 1520 kg/m^3^, showing a much higher specific strength than conventional high-strength concrete. Wang et al. [13] further optimized the mixture design and produced lightweight ultra-high-performance concrete with a density of 1890 kg/m^3^ and a compressive strength as high as 134 MPa, confirming the potential of HGMs for achieving both ultra-high strength and low weight. Li et al. [14] developed high-strength lightweight cementitious composites with HGMs and found that when 60% HGMs were incorporated, the density decreased to about 970 kg/m^3^ while the compressive strength remained around 31 MPa, achieving an optimal structural efficiency at 30% replacement. Kim et al. [15] developed high-strength lightweight cementitious composites using HGMs in a low water-to-cement matrix; after ambient and heat curing, the 28-day compressive strengths reached 69 MPa and 97 MPa, respectively, with densities below 1.5 g/cm^3^. Wang et al. [16] further studied the influence of HGMs on the performance of lightweight ultra-high-performance concrete and the optimization of mix proportions, providing a new approach for synergizing ultra-high performance and lightness.

Orthogonal experimental design has been widely adopted for multi-factor optimization of concrete mix proportions [17]. This method allows the effects of several factors to be evaluated with a limited number of test runs and the determination of the most influential parameters. In the field of lightweight aggregate concrete, Yang et al. [18] used a three-factor, three-level orthogonal design combined with range analysis and variance analysis to quantify the effects of water–cement ratio, steel slag replacement ratio and ceramsite replacement ratio on the fluidity and compressive strength of steel slag-ceramsite foam concrete; they found that water–cement ratio had a greater influence on compressive strength than ceramsite replacement, while steel slag replacement had the most significant effect on fluidity. For EPS lightweight structural concrete, researchers used orthogonal design coupled with machine learning to systematically analyze the effects of EPS content, water–binder ratio and POM fiber content on compressive strength, splitting tensile strength, thermal conductivity and freeze–thaw resistance, revealing that EPS content most significantly enhanced freeze–thaw and thermal insulation performance [19]. Studies on the application of orthogonal test methods in the mix proportion design of recycled lightweight aggregate concrete have also shown that water–binder ratio, fly ash content, sand ratio and recycled aggregate content all significantly influence compressive strength, and range analysis can effectively identify the degree of influence of each factor [20].

Expanded clay ceramsite is a traditional lightweight aggregate produced by sintering natural clay at high temperatures; its characteristic honeycomb-like internal structure provides good thermal insulation, chemical stability and fire resistance [21]. Durability studies have shown that ceramsite lightweight aggregate concrete exhibits complex performance degradation under freeze–thaw cycles because of its high porosity, and the combined use of ceramsite and natural aggregates can achieve density adjustment while maintaining structural strength and increasing the freeze–thaw durability coefficient by an average of 124% [22]. Regarding the engineering application of waste glass powder, flexural performance tests on lightweight aggregate concrete beams with high-volume waste glass powder replacing cement showed that beams with 20% waste glass powder exhibited a 12.0% increase in initial flexural stiffness and a 9.2% increase in ductility, while the density decreased by 18.7%, confirming the feasibility of using waste glass powder in structural lightweight concrete members [23]. For mitigation of ASR in waste glass aggregate concrete, Gholampour et al. [24] investigated the effect of slag–fly ash composite binders on suppressing ASR expansion in glass–sand concrete; they found that fly ash was superior to slag in reducing ASR expansion, and a combination of 20% slag and 30% fly ash resulted in mechanical properties comparable to natural sand concrete. A recent comprehensive review further pointed out that the effectiveness of ASR mitigation strategies ranks as follows: silica fume (10% replacement) > fly ash (30% replacement) > slag (60% replacement). Glass particles that are finer than 300 μm exhibit beneficial pozzolanic behavior, while particles coarser than 1 mm show high reactivity; particle size distribution is a key factor controlling ASR risk [25].

Recycled glass pumice (microcellular glass pumice aggregate, MGPA), a novel artificial porous lightweight aggregate produced by foaming waste glass at high temperature, has recently attracted considerable attention due to its combination of lightness, high strength and environmental friendliness [26]. Multi-scale glass-based material design has been shown to achieve high compressive strength with extremely low cement content, providing new ideas for the application of glass-based materials in precast and modular construction [27]. High-strength fly ash ceramsite lightweight aggregate concrete (cylinder compressive strength > 20 MPa) has been extended to cover a wide range of compressive strengths from 20 to 110 MPa, elastic moduli from 10 to 45 GPa, and apparent densities from 1250 to 2350 kg/m^3^, indicating that recycled glass pumice has good potential for similar performance ranges [28]. The introduction of hollow glass microspheres has endowed lightweight cementitious composites with synergistic ultra-high strength and low density, demonstrating the great potential of glass-based materials for high-strength lightweight applications [12,13,15]. Moreover, the systematic use of orthogonal test methods in lightweight aggregate concrete mix proportion optimization has been proven to effectively identify the primary and secondary order of factors and optimize design parameters [17,19,20].

Although ceramsite has been extensively studied as a conventional lightweight aggregate [21], the use of recycled glass pumice as a novel green aggregate that can partially or completely replace ceramsite, and its systematic comparison with ceramsite, remains limited [29]. Most existing studies have focused on single types of lightweight aggregates, and systematic comparisons under the same experimental framework are still scarce. This study takes recycled glass pumice and ceramsite as research objects, adopts an orthogonal experimental design with four factors and five levels, systematically investigates the effects of water–cement ratio, cement content, coarse MGPA replacement ratio and fine MGPA replacement ratio on the dry density and compressive strength of lightweight aggregate concrete, and compares the mechanical properties of the two types of concrete. The results provide an experimental basis for the resource utilization of waste glass and the engineering application of lightweight aggregate concrete. It should be noted that the present study focuses exclusively on short-term mechanical properties; durability performance, including alkali–silica reaction (ASR) resistance, is not investigated and is identified as a key direction for future research.

## 2. Materials and Methods

### 2.1. Materials

#### 2.1.1. Cementitious Materials

Ordinary Portland cement P·O 42.5 was used throughout the study. Its physical properties were as follows: density 3080 kg/m^3^, specific surface area 335 m^2^/kg, initial and final setting times 160 min and 327 min, 3-day compressive strength 25.42 MPa and flexural strength 5.36 MPa, 28-day compressive strength 52.70 MPa and flexural strength 8.40 MPa. Class II fly ash from a thermal power plant was used as a supplementary cementitious material; its density was 2290 kg/m^3^, water demand ratio 103%, loss on ignition 7.5%, moisture content 0.7%, SO_3_ 1.1% and free CaO 0.8%. The main chemical compositions of the OPC were: SiO_2_ 21.5%, Al_2_O_3_ 5.3%, Fe_2_O_3_ 3.8%, CaO 62.1%, MgO 2.6%, and SO_3_ 2.4%. The Class II fly ash (classified according to GB/T 1596-2017) [30] had a loss on ignition of 7.5%, water demand ratio of 103%, SO_3_ content of 1.1%, and free CaO content of 0.8%, all meeting the requirements of GB/T 1596-2017 for Class II fly ash.

In this study, the water–cement ratio (w/c) is calculated based on the mass of Portland cement only, excluding fly ash, following the convention of the orthogonal design. This choice was made to maintain direct comparability with the ceramsite reference mixture, in which fly ash was also used as a supplementary cementitious material. However, it is acknowledged that the water-to-binder ratio (w/b), including fly ash, would be more appropriate for describing the effective water content in the cementitious system; this limitation is considered in the interpretation of the results.

#### 2.1.2. Glass Lightweight Aggregates (MGPA)

**Figure 1:**
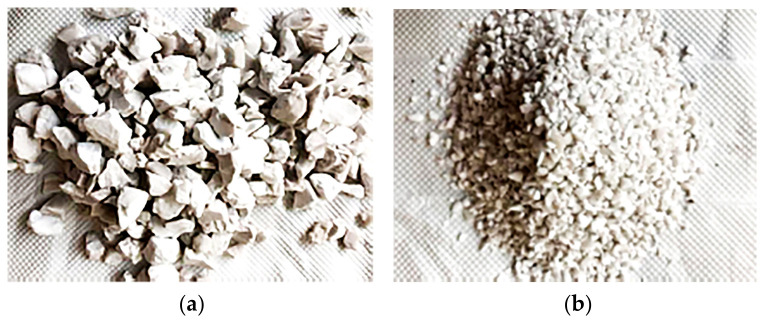
Appearance of the MGPA: (**a**) coarse fraction (5–15 mm) and (**b**) fine fraction (0–5 mm).

The MGPA was a milky white porous material produced by grinding waste glass, mixing with additives, and sintering at high temperature until foaming occurred (Figure 1). Two size fractions were used: coarse MGPA (5–15 mm) and fine MGPA (0–5 mm). Their physical and mechanical properties are summarized in Table 1. The fine MGPA had a bulk density of 566 kg/m^3^, apparent density 803 kg/m^3^, voidage 29.5% and 24 h water absorption 9.5%. The coarse MGPA had a bulk density of 312 kg/m^3^, apparent density 668 kg/m^3^, voidage 45.8%, 24 h water absorption 18.1% and cylinder compressive strength 3.36 MPa. The much lower density of MGPA compared to natural aggregates is the main reason for its ability to produce very lightweight concrete.

#### 2.1.3. Expanded Clay Ceramsite

Expanded clay ceramsite was used as a reference lightweight aggregate. Its particle size was 5–16 mm, bulk density 678 kg/m^3^ (density grade 700), apparent density 1026 kg/m^3^, voidage 33.9%, 1-h water absorption 13.4%, and cylinder compressive strength 3.8 MPa.

#### 2.1.4. Other Materials

Natural river sand (fineness modulus 2.6) was used as fine aggregate, crushed stone (5–20 mm) as normal coarse aggregate, and a polycarboxylate superplasticizer was used to adjust workability.

### 2.2. Orthogonal Experimental Design

Four factors were investigated: water–cement ratio (WR), cement content (C), replacement ratio of fine MGPA (FA) and replacement ratio of coarse MGPA (CA). The levels of each factor are given in Table 2. Water–cement ratio had four levels (0.40, 0.42, 0.44, 0.46); cement content also had four levels (330, 340, 350, 360 kg/m^3^); the replacement ratios of both fine and coarse MGPA had five levels (0%, 25%, 50%, 75%, 100%). According to the orthogonal design principle, 20 mixture proportions were prepared, designated S1 to S20. The detailed mix proportions are listed in Table 3.

It should be noted that in this orthogonal design (see Table 2), the water–cement ratio (WR) and cement content (C) are perfectly negatively correlated (WR = 0.40 with C = 360 kg/m^3^, WR = 0.42 with C = 350 kg/m^3^, WR = 0.44 with C = 340 kg/m^3^, and WR = 0.46 with C = 330 kg/m^3^). Statistically, this causes complete confounding between these two factors, making it impossible to separate their individual effects by range analysis. Consequently, the identical R values for WR and C in Table 4 and Table 5 are mathematically unavoidable and do not indicate equal contributions to performance. To address this limitation, we treat WR and C as a combined coupled factor in the subsequent discussion and refrain from ranking their independent influences in the conclusions.

It should also be noted that the mixing water reported in Table 3 is the total water added to the mixture. No additional water was added to compensate for the water absorption of MGPA, nor were the aggregates pre-saturated prior to mixing. The high water absorption of MGPA (18.1% for coarse and 9.5% for fine fraction, as shown in Table 1) means that a portion of the mixing water was absorbed by the aggregates during mixing, reducing the effective water available for cement hydration. Consequently, the effective water–cement ratio in MGPA mixtures is lower than the nominal values listed in Table 3. This effect is more pronounced at higher MGPA replacement ratios and should be considered when interpreting the strength results—particularly for mixtures with 100% coarse MGPA replacement, where the actual effective w/c may be substantially lower than the nominal value. The polycarboxylate superplasticizer was added at a fixed dosage of 4.2 kg/m^3^ (approximately 1.0% by mass of total binder) for all MGPA mixtures to maintain adequate workability. The dosage was kept constant across all 20 orthogonal groups to isolate the effects of the four investigated factors.

**Figure 2:**
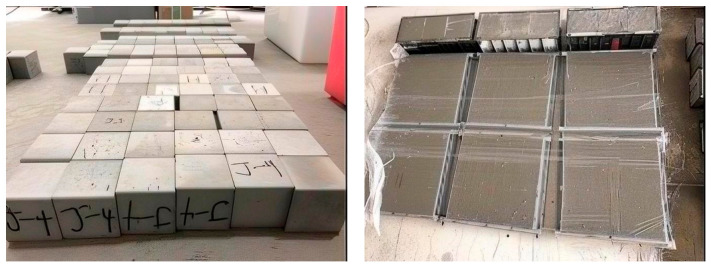
Cast concrete cube specimens before curing.

For each mixture, three 150 mm × 150 mm × 150 mm cubes were cast. Figure 2 shows the prepared specimens. All specimens were demolded after 24 h and then cured in a standard curing room (20 ± 2 °C, RH ≥ 95%) for 7 d and 28 d before testing.

### 2.3. Testing Methods

#### 2.3.1. Dry Density

The dry density was determined according to GB/T 17431.2-2010 [31]. The cubes were dried in an oven at 105 °C until constant mass, then cooled and weighed. The density was calculated as

ρ = m/V.(1)

#### 2.3.2. Compressive Strength

Compressive strength tests were performed using a 3000 kN compression testing machine (Shanghai Hualong Testing Instruments Co., Ltd., Shanghai, China) at a loading rate of 400 kN/min. The strength was calculated as

fcu = P/A.(2)

#### 2.3.3. Range Analysis

Range analysis was used to process the orthogonal data. For each factor at a given level, the sum of test results (Kj), the average (kj) and the range R = max(kj) − min(kj) were calculated. A larger R indicates a stronger influence of that factor on the response.

### 2.4. Ceramsite Concrete Reference Mixture

To provide a benchmark for comparison, a separate set of tests was performed on ceramsite lightweight aggregate concrete with fixed water–cement ratio (0.45) and three ceramsite replacement ratios (15%, 20% and 25% by mass). The mixtures are detailed in Table 4. The 28-day compressive strength and dry density of each mixture were measured. The mixture with 20% ceramsite was selected as the optimal reference, giving a strength of 20.5 MPa and a dry density of 1710 kg/m^3^.

The superplasticizer dosage for the ceramsite concrete mixtures was 4.76 kg/m^3^ (approximately 1.06% by mass of total binder), as listed in Table 4. This dosage was determined based on preliminary trials to achieve a target slump of approximately 160–180 mm.

## 3. Results and Discussion

### 3.1. Orthogonal Test Results of MGPA Concrete

The measured dry density and compressive strength of the 20 MGPA concrete mixtures are presented in Table 5.

**Figure 3:**
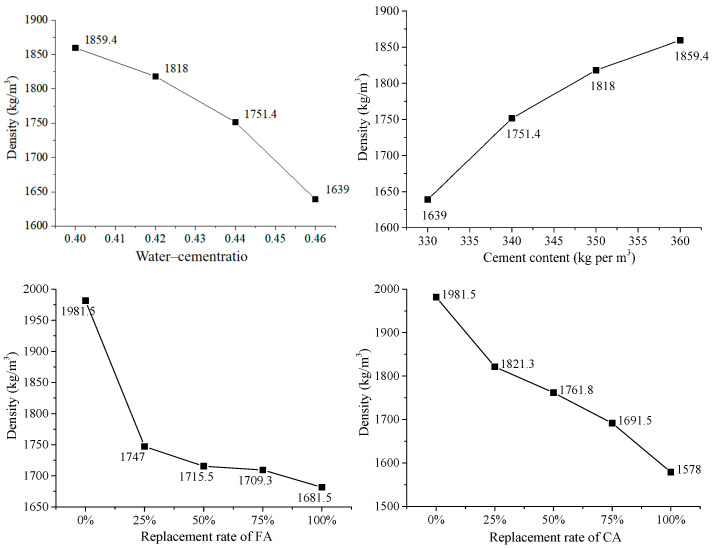
Main-effect plots of dry density from the range analysis (level means for each factor).

**Figure 4:**
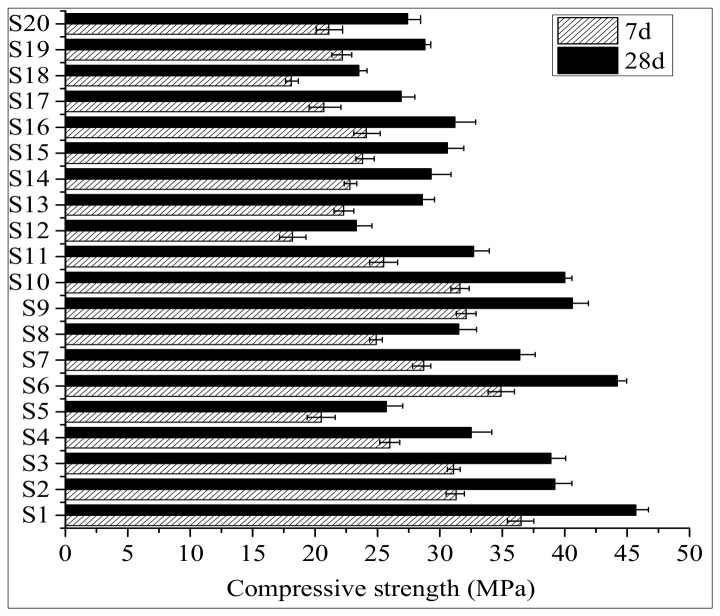
The 7-d and 28-d compressive strengths of the MGPA concrete samples.

The dry density decreased sharply with increasing MGPA replacement ratio(Figure 3). The lowest dry density (1445 kg/m^3^) was obtained for mixture S19, with WR = 0.46 and cement = 330 kg/m^3^. This value is 46% lower than that of the reference ordinary concrete (S1, 2089 kg/m^3^). For all mixtures, the 7-day compressive strength reached 77–80% of the 28-day strength(Figure 4), indicating rapid early strength development. This is because the hydration of cement and fly ash was not yet complete at 7 d, resulting in a lower bond strength; with continued hydration, the interfacial transition zone became denser and the strength increased.

It should be noted that the range analysis employed in this study is a standard method for orthogonal design optimization and has been widely applied in concrete mix proportion studies [17,19,20]. The consistent trends observed across the 20 mixtures, together with the generally close values among replicates, indicate that the variability in the test data does not affect the reliability of the factor ranking and optimization. The consistent trends provide reliable directional guidance for identifying the primary factors influencing dry density and compressive strength. It should also be noted that slump tests were not performed for the MGPA concrete mixtures, as the primary objective of this study was to evaluate dry density and compressive strength under the orthogonal design framework; workability characterization remains a direction for future research. The fixed superplasticizer dosage (4.2 kg/m^3^) was adopted based on preliminary trials to ensure adequate workability for specimen casting across all MGPA mixtures, but slump values were not recorded as workability was not a variable in the orthogonal design. In addition, standard deviations and coefficients of variation are not reported in this study, as the range analysis in orthogonal design optimization is based on mean values of each mixture for factor ranking and identification of primary and secondary order. The consistent trends observed across the 20 mixtures provide sufficient confidence in the reliability of the results. Future studies will include detailed statistical analysis of data variability (e.g., SD, CV, and error bars) to further strengthen the conclusions.

### 3.2. Range Analysis of Dry Density

Table 6 summarizes the range analysis for dry density. The R values show that the influencing factors rank as: coarse aggregate replacement (R = 402.7) > fine aggregate replacement (300.0) > water–cement ratio (220.4) = cement content (220.4). Coarse aggregate replacement is the most significant factor because MGPA has an extremely low bulk density (312 kg/m^3^) compared to natural gravel (≈1500 kg/m^3^). Increasing the coarse MGPA content from 0% to 100% reduced the dry density from 1981.5 kg/m^3^ to 1578.8 kg/m^3^, a decrease of 20.3%. For fine aggregate replacement, the density decreased from 1981.5 kg/m^3^ to 1681.5 kg/m^3^ (15.1%). Notably, the density reduction was most pronounced when FA increased from 0% to 25%, accounting for the majority of the total decrease. This is attributed to the fact that at 25% replacement, the fine MGPA particles begin to replace a sufficient volume of the denser natural sand, creating a more porous fine aggregate skeleton without yet being offset by the filling effect of the finer MGPA particles. At higher FA levels, the additional density reduction becomes less steep because the remaining natural sand is gradually replaced, and the increasing amount of fine MGPA particles may partially fill the voids between coarse aggregates, moderating the overall density decrease. Water–cement ratio had a smaller influence because higher water–cement ratios increase internal porosity, thereby lowering density. Cement content increases the proportion of the denser cement paste, thus increasing density. The range analysis presented in Table 6 and Table 7 follows the standard orthogonal design procedure, in which the range R = max(kj) − min(kj) is used to rank the influence of each factor. A larger R indicates a stronger influence on the response.

According to BS EN 206-1 [32], lightweight concrete is defined as having an oven-dry density of not less than 800 kg/m^3^ and not more than 2000 kg/m^3^. On this basis, mixtures S1 (2089 kg/m^3^) and S6 (2041 kg/m^3^) exceed the upper limit and do not qualify as lightweight concrete, whereas the remaining 18 mixtures, with densities ranging from 1445 to 1938 kg/m^3^, fall within the lightweight concrete category.

### 3.3. Range Analysis of Compressive Strength

The range analysis for 7-d and 28-d compressive strength is given in Table 7. At 28 d, the R values rank as: CA (11.13) > WR (10.98) = C (10.98) > FA (8.63). At 7 d, WR and C had larger R (9.20) than CA (8.80), indicating that the early strength is dominated by the hydration of cementitious materials, while the aggregate skeleton becomes more important after 28 d. Increasing the coarse MGPA replacement from 0% to 100% reduced the 28-day strength from 38.45 MPa to 27.33 MPa. The water–cement ratio showed an optimum value (0.42), above or below which the strength decreased. Cement content had an optimum at 350 kg/m^3^. Fine MGPA replacement caused a sharp drop when increased from 0% to 25% (16.4% loss), but thereafter the strength fluctuated only slightly; a small recovery was observed at 50% replacement. The optimum mixture (highest 28-day strength, 40.0 MPa) was S10 (WR = 0.42, C = 350 kg/m^3^, FA = 50%, CA = 50%).

When interpreting the range analysis results in Table 6 and Table 7, the complete confounding between WR and C noted in Section 2.2 must be recalled. Since the R values are identical, it cannot be concluded that water–cement ratio and cement content have equal effects on density or strength. Their respective true effects can only be determined by future factorial-design or single-factor experiments. Within the framework of the present data, it is more rigorous to treat WR and C as a combined factor. Accordingly, we do not rank WR and C independently.

In this study, three replicate specimens were cast for each mixture, and the results showed generally consistent trends across all test groups. The range analysis effectively identified the relative importance of the four factors. Among them, coarse aggregate replacement (CA) consistently exhibited the largest influence on both dry density and compressive strength, while fine aggregate replacement (FA) showed the smallest effect. These trends are consistent with the physical mechanisms discussed in the following sections. While range analysis is a standard method for orthogonal design optimization, it does not provide significance testing; the results should therefore be interpreted as an exploratory assessment of factor influences.

### 3.4. The Effect of Water–Cement Ratio on Compressive Strength

**Figure 5:**
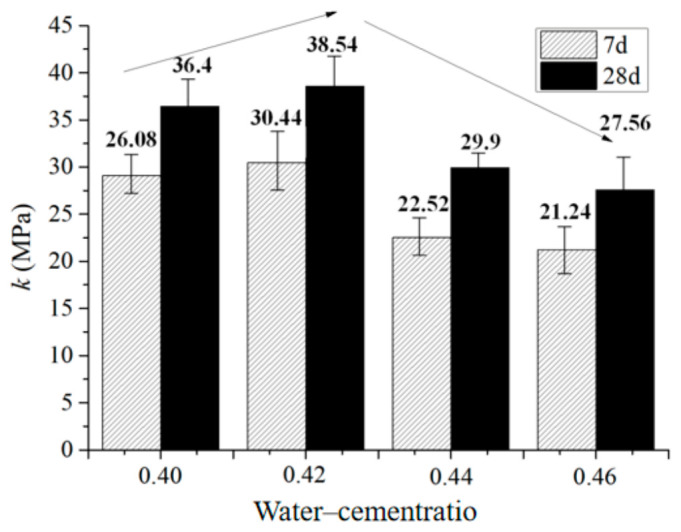
The effect of water–cement ratio on the compressive strength of the MGPA concrete samples.

Figure 5 shows that the 28-day strength increased from 36.40 MPa (WR = 0.40) to a maximum of 38.54 MPa (WR = 0.42) and then decreased to 27.56 MPa (WR = 0.46). A low water–cement ratio (0.40) leads to a thick, poorly fluid paste that cannot uniformly coat the aggregates, resulting in weak interfaces and lower strength. At WR = 0.42, the paste has good fluidity and sufficient hydration, leading to dense interfacial transition zones and high strength. When WR exceeds 0.42, the paste becomes too dilute; during vibration the heavy cement paste flows downwards, leaving the upper part of the specimen poorly bonded, thus reducing strength.

### 3.5. The Effect of Cement Content on Compressive Strength

**Figure 6:**
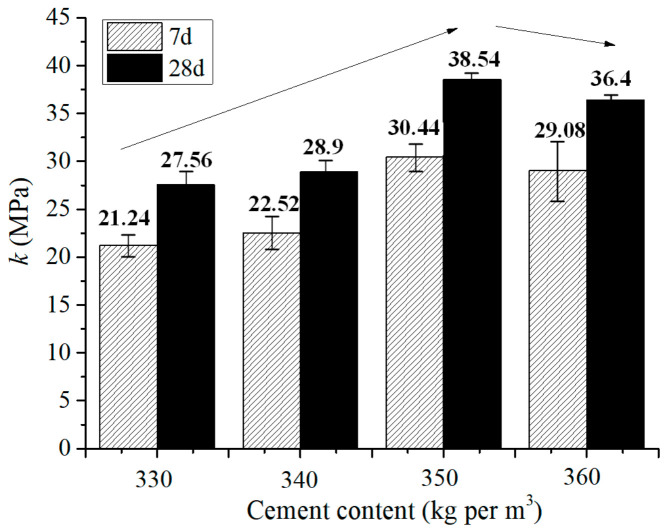
The effect of cement content on the compressive strength of the MGPA concrete samples.

Figure 6 illustrates that the 28-day strength rose sharply from 27.56 MPa (330 kg/m^3^) to 38.54 MPa (350 kg/m^3^), a 39.8% increase, and then fell to 36.40 MPa (360 kg/m^3^). Increasing cement content initially increases the amount of cementitious material, which improves bonding between aggregates and fills voids, raising strength. However, to keep a constant water–cement ratio, increasing cement content also requires more water. This is because the water amount in each mixture is directly calculated by multiplying the water–cement ratio by the cement content. Therefore, when the cement content is increased while maintaining a constant w/c, the absolute quantity of mixing water must increase proportionally. This additional water, however, does not contribute positively to the strength development. Instead, it increases the overall porosity of the hardened cement paste after evaporation, dilutes the concentration of cementitious materials in the paste, and reduces the viscosity of the paste film coating the aggregate surfaces. As a result, the interfacial transition zone between the paste and the aggregates becomes less dense and more porous, which weakens the bond between the matrix and the aggregates and ultimately leads to a reduction in compressive strength. In other words, when the adverse effect of the extra water on the paste quality outweighs the beneficial effect of the additional cementitious material, the strength begins to decrease. This trade-off explains the observed peak strength at a moderate cement content (350 kg/m^3^) in this study. When the adverse effect of the extra water outweighs the benefit of extra cement, the strength decreases. It should be noted that the water–cement ratios discussed here are nominal values based on total mixing water. Due to the high water absorption of MGPA, the effective water–cement ratio in the cement paste is lower than the nominal value, particularly for mixtures with high MGPA content. This absorption effect may partially explain why the optimal nominal w/c for MGPA concrete (0.42) differs from that of conventional concrete.

### 3.6. The Effect of Fine MGPA Replacement on Compressive Strength

**Figure 7:**
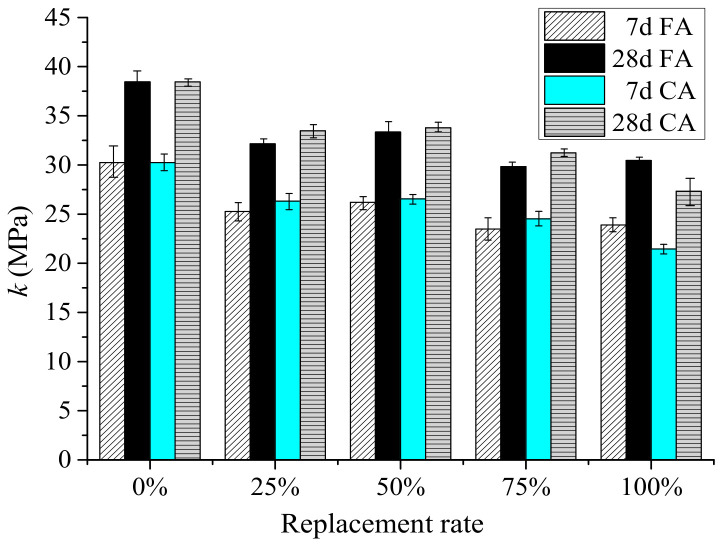
The effect of the fine MGPA replacement ratio on compressive strength.

The 28-day strength decreased by 16.4% when FA increased from 0% to 25% (Figure 7). In the range 25–100%, the strength fluctuated within ±10.6%, with a slight recovery at 50% (from 32.16 MPa to 33.35 MPa). The initial sharp drop is due to the inherent pores and microcracks in the MGPA particles, which act as initial damage sites. At 50% fine MGPA replacement, the high water absorption of MGPA may reduce the effective water–cement ratio at the interface, potentially leading to denser hydration products and improved bonding, which could partially compensate for the strength loss. However, this interpretation remains speculative without direct microstructural evidence. Thus, 50% is the recommended optimal fine MGPA replacement.

### 3.7. The Effect of Coarse MGPA Replacement on Compressive Strength

When CA increased from 0% to 25%, the 28-day strength fell from 38.45 MPa to 33.48 MPa (12.9% loss). Between 25% and 50%, a slight increase (33.48 → 33.78 MPa) occurred, followed by further decreases at 75% and 100% (31.23 MPa and 27.33 MPa, respectively). The dominant negative effect is the low strength of the MGPA particles (cylinder compressive strength 3.36 MPa vs. >30 MPa for natural gravel). The positive effect is the rough, angular shape and high porosity of MGPA, which may improve mechanical interlocking and water absorption, potentially leading to a stronger interface. Direct microstructural observations would be needed to confirm this mechanism. At 25–50% replacement, the positive effect nearly compensates for the negative effect, giving a small strength recovery. The optimal coarse MGPA replacement is 50%.

### 3.8. Failure Mode Analysis

**Figure 8:**
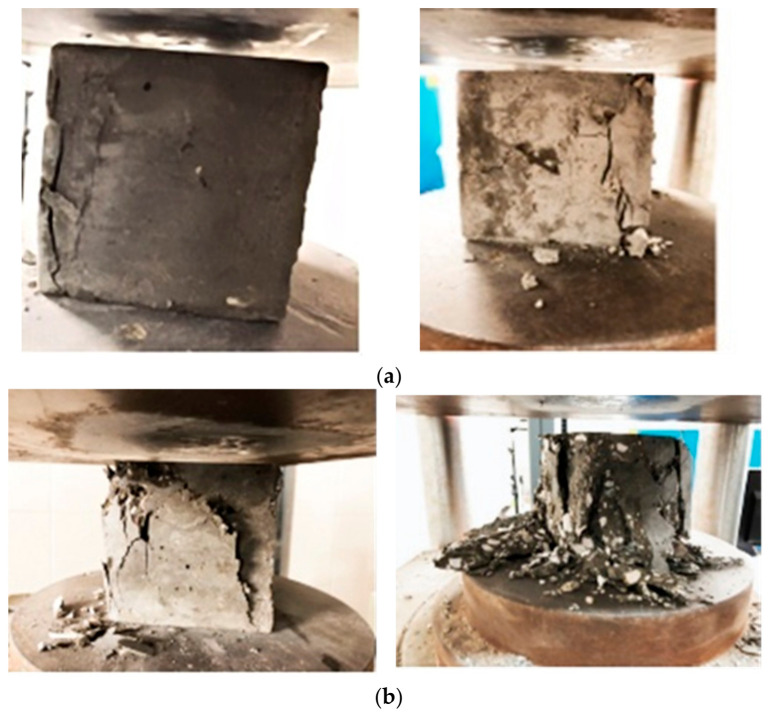
Failure modes of (**a**) ordinary concrete and (**b**) MGPA concrete.

Figure 8 shows the failure process of ordinary concrete cubes and MGPA concrete cubes. Ordinary concrete fails through the development of microcracks along the aggregate–paste interfaces, with the aggregates themselves remaining largely intact. For MGPA concrete with a high replacement ratio, failure occurred suddenly without noticeable warning; the cracks propagated rapidly from the top to the bottom of the cube, accompanied by a sharp sound. Post-failure observation revealed that failure occurred both along the interface and through the MGPA particles, which were often split into two halves. This indicates that at high replacement ratios, the strength of the MGPA particles themselves becomes the limiting factor for the concrete compressive strength.

For mixtures with low MGPA replacement ratios (e.g., S1 and S6, with 0% coarse MGPA), failure was initiated by microcracks developing along the aggregate–paste interface, and the cracks propagated in a relatively stable manner before reaching the peak load. The aggregates remained largely intact after failure, indicating that the interfacial bond was the weakest link. In contrast, for mixtures with high MGPA replacement ratios (e.g., S5 and S19, with 100% coarse MGPA), failure occurred suddenly without noticeable warning. Cracks propagated rapidly from the top to the bottom of the cube, accompanied by a sharp audible sound. Post-failure observation revealed that failure occurred both along the interface and through the MGPA particles, which were often split into two halves. This indicates that at high replacement ratios, the strength of the MGPA particles themselves becomes the limiting factor for the concrete compressive strength. The transition from interfacial failure to aggregate failure with increasing MGPA content is consistent with the strength reduction observed in the range analysis.

### 3.9. Stress–Strain Behavior of MGPA Concrete

The stress–strain response of MGPA concrete, inferred from the recorded peak load and the observed failure characteristics, exhibits a more brittle behavior with increasing MGPA replacement ratio. At low replacement levels, the descending branch of the stress–strain curve is relatively gradual, reflecting the contribution of the natural aggregates to post-peak load resistance. At high MGPA replacement ratios, the stress drops sharply after reaching the peak, indicating that the porous glass aggregate provides limited resistance to crack propagation. This observation aligns with the sudden failure mode described above for high-replacement mixtures, and is consistent with the lower cylinder compressive strength of the MGPA particles (3.36 MPa) compared to natural gravel.

### 3.10. Chemical Aspects of the Interfacial Transition Zone

From a chemical perspective, the performance of MGPA concrete is also influenced by the pozzolanic reactivity of the glass phase in the alkaline cementitious environment. The surface of MGPA particles contains amorphous silica, which can react with calcium hydroxide (CH) released during cement hydration to form calcium-silicate-hydrate (C-S-H) gel at the interfacial transition zone (ITZ). This pozzolanic reaction densifies the ITZ and enhances the bond between the MGPA particles and the cement paste, partially offsetting the mechanical weakness of the porous glass aggregate. However, the extent of this chemical contribution is limited by the relatively low reactivity of the glass phase at ambient temperature over the 28-day curing period. The presence of fly ash further consumes CH through its own pozzolanic reaction, competing with the glass aggregate for CH. Overall, the net effect of these chemical processes on mechanical performance appears to be secondary compared with the physical effects of aggregate density and porosity, which is consistent with the dominant influence of coarse MGPA replacement ratio observed in the range analysis.

## 4. Comprehensive Comparison Between MGPA Concrete and Ceramsite Concrete

Before presenting the comparison, it is important to clarify that the ceramsite concrete and MGPA concrete were not tested under perfectly equivalent conditions. The ceramsite concrete was evaluated at a fixed water–cement ratio of 0.45 with only three ceramsite replacement ratios (15%, 20%, and 25%) and was not subjected to the same orthogonal optimization as the MGPA system. The two systems also differ in total binder content (ceramsite: 450 kg/m^3^; MGPA: approximately 390–430 kg/m^3^) and water–cement ratio (0.45 vs. 0.42 in the optimum MGPA mix). The primary purpose of this comparison is to assess the potential of MGPA relative to a widely used conventional material under respectively optimized mix designs, rather than to isolate the aggregate effect through a strictly controlled factorial comparison. This approach is considered appropriate for an initial feasibility study; however, a strictly controlled comparison with matched binder content, water–cement ratio, and admixture dosage, with both systems optimized under the same criterion, is needed in future work to enable a more rigorous attribution of performance differences to aggregate type.

In addition, this study does not include any durability testing, particularly regarding alkali–silica reaction (ASR), which is the primary durability concern for glass-based aggregates [10,11,24,25]. Class II fly ash was used in all mixtures at a replacement ratio of approximately 15–17.5% of the total binder, and previous studies have demonstrated that fly ash can effectively mitigate ASR expansion [24,25]; however, the effectiveness of this specific dosage for MGPA requires further experimental validation. Until ASR expansion data are available, MGPA should be used with caution—preferably in non-exposed, non-structural, or indoor precast applications.

Furthermore, the mixing water in this study was not adjusted for MGPA absorption, meaning the effective water–cement ratio varies with MGPA replacement ratio. This adds another variable to the MGPA–ceramsite comparison and further limits the direct attribution of performance differences to aggregate type. In addition, workability (slump) was not evaluated for MGPA concrete in this study, and the superplasticizer dosage was not optimized. Future studies should include workability characterization to facilitate practical application. It is acknowledged that workability is an important property for concrete performance, particularly for high-absorption aggregates such as MGPA. However, the present orthogonal design focused on dry density and compressive strength as the primary response variables, while workability was maintained at an acceptable level for casting by using a fixed superplasticizer dosage. A systematic evaluation of workability across different mixture proportions, including slump or flow measurements, is therefore beyond the scope of this initial study and will be addressed in future research.

### 4.1. Physical and Mechanical Properties of the Aggregates

Table 8 compares the key properties of the two lightweight aggregates. The bulk density of coarse MGPA is only 46% of that of ceramsite, giving MGPA a much greater potential for weight reduction. However, the water absorption of MGPA (18.1%) is higher than that of ceramsite (13.4%), because the foaming process creates interconnected pores. This higher absorption must be taken into account by pre-wetting the aggregate or adding extra water to the mix.

### 4.2. Comparison of Optimum Mix Proportions and Mechanical Properties

The optimum mix proportions and corresponding properties are compared in Table 9. The optimum MGPA concrete (S10) had a dry density of 1788 kg/m^3^, only 4.6% higher than that of the ceramsite reference, while its 28-day compressive strength reached 40.0 MPa, substantially higher than the 20.5 MPa recorded for the ceramsite mixture under the conditions tested. The specific strength (strength/density) of MGPA concrete was 22.4 kN·m/kg, about 1.87 times that of ceramsite concrete (12.0 kN·m/kg). Even the fully replaced MGPA concrete (S19) gave a dry density of 1445 kg/m^3^ and a 28-day strength of 28.8 MPa, which still satisfies the LC25 grade for structural lightweight concrete and provides a specific strength of 19.9 MPa·m^3^/kg, much higher than that of ceramsite concrete.

While the comparison is not fully equivalent due to the differences in mix design noted above, the superior performance of MGPA concrete in this study may be attributed to two factors: (1) the rough surface and high water absorption of MGPA may improve the interfacial bond with the cement paste, potentially producing a denser interfacial transition zone; (2) the optimized water–cement ratio and cement content in the orthogonal design appear to promote more complete hydration of the cementitious materials, further strengthening the matrix. It should be noted, however, that these interpretations are speculative in the absence of direct microstructural evidence (e.g., SEM, MIP, or optical microscopy), and should be treated as hypotheses requiring future verification.

### 4.3. Feasibility of Replacing Ceramsite with MGPA

The early strength development of MGPA concrete (77–80% of 28-day strength at 7 d) is similar to that of ceramsite concrete, which is favorable for fast construction. For lightweight applications, MGPA is far superior because its own bulk density is only 46% of that of ceramsite; the fully replaced MGPA concrete (S19) has a dry density of 1445 kg/m^3^, which is 15.5% lower than the ceramsite concrete density (1710 kg/m^3^). For structural applications, the optimum MGPA concrete offers a 28-day compressive strength of 40.0 MPa, which can easily satisfy the requirements for structural lightweight concrete (LC25 to LC40). Within the scope of this study, MGPA shows promise as an alternative to ceramsite, especially for applications where both weight reduction and high strength are desired. However, a strictly fair comparison—with matched binder content, water–cement ratio, and admixture dosage, and with both systems optimized under the same criterion—remains to be conducted in future work. In practice, the replacement ratio can be selected according to the specific requirements: for maximum lightness, use 100% replacement (S19); for maximum strength, use 50% replacement (S10); intermediate replacements can achieve a compromise.

Regarding practical applications, MGPA concrete with 50% replacement of both fine and coarse aggregates (mixture S10) achieves a compressive strength of 40.0 MPa and a dry density of 1788 kg/m^3^, which satisfies the requirements for structural lightweight concrete (LC25–LC40) and is suitable for precast components such as non-load-bearing walls, partition panels, and lightweight floor systems. For applications where weight reduction is prioritized over strength, the fully replaced mixture (S19, 1445 kg/m^3^, 28.8 MPa) offers a viable option for thermal insulation layers and filler applications. From an economic perspective, MGPA is manufactured from waste glass through an energy-intensive sintering process, which may result in higher production cost than ceramsite, but offers environmental benefits by diverting glass from landfills and reducing natural clay consumption.

Several limitations of the present work should be acknowledged. Fracture resistance was not evaluated. The mechanistic interpretations regarding aggregate-paste bonding and ITZ densification are based on indirect evidence from mechanical testing and macroscopic observation, without microstructural investigations (e.g., SEM, MIP, or optical microscopy). In addition, the conclusions are based on range analysis without statistical significance testing; ANOVA will be performed in future work to verify the statistical validity of the observed differences. These limitations are all identified as directions for future research.

## 5. Conclusions

(1) The coarse MGPA has a bulk density of 312 kg/m^3^ (only 46% of that of ceramsite), a cylinder compressive strength of 3.36 MPa, and a 24-h water absorption of 18.1%. The fine MGPA has a bulk density of 566 kg/m^3^, an apparent density of 803 kg/m^3^, a voidage of 29.5% and a water absorption of 9.5%. All properties meet the requirements of GB/T 17431.2-2010 [31].
(2) The factors influencing the dry density of MGPA concrete rank as: coarse aggregate replacement > fine aggregate replacement > water–cement ratio = cement content. The lowest dry density (1445 kg/m^3^) was obtained for mixture S19 (WR = 0.46, cement = 330 kg/m^3^, 100% coarse MGPA replacement and partial fine MGPA replacement).
(3) Owing to the complete confounding between WR and C, their individual effects on 28-day compressive strength cannot be distinguished. After acknowledging this limitation, the range analysis indicates that coarse aggregate replacement (CA) has the greatest influence on strength, fine aggregate replacement (FA) has the least, while WR and C should be treated as a coupled factor rather than ranked independently.
(4) The 7-day compressive strength of MGPA concrete reaches 77–80% of the 28-day strength, indicating rapid early strength development. The optimal fine and coarse MGPA replacement ratios are both 50%, which balance lightness and strength.
(5) Under the specific mix designs tested in this study, the optimal MGPA concrete showed only 4.6% higher dry density than the ceramsite reference, but achieved substantially higher 28-day compressive strength and specific strength. However, this comparison is preliminary, as the two systems were not optimized under identical conditions.
(6) Subject to the limitation that long-term durability (particularly ASR resistance) has not been verified in this study, recycled glass pumice shows promise as a substitute for ceramsite in lightweight aggregate concrete. The replacement ratio can be chosen according to design requirements—full replacement for maximum lightness, and 50% replacement for maximum strength—but durability validation is recommended before use in structural or exposed precast components.

A limitation of this study is that the ceramsite concrete was not optimized through the same orthogonal design procedure as the MGPA concrete. Future studies should compare the two aggregate systems under matched binder content, water–cement ratio, and admixture dosage, with both systems optimized under the same criterion, to enable a more rigorous attribution of performance differences to aggregate type. In addition, it is acknowledged that the present study relies on range analysis without significance testing, as is common in many orthogonal design optimization studies in this field. The trends identified herein provide reliable directional guidance for further research and engineering practice.

## Figures and Tables

**Table 1:** Physical and mechanical properties of the MGPA.

Type	Bulk Density (kg/m^3^)	Apparent Density (kg/m^3^)	Voidage (%)	24 h Water Absorption (%)	Cylinder Compressive Strength (MPa)
Fine (0–5 mm)	566	803	29.5	9.5	—
Coarse (5–15 mm)	312	668	45.8	18.1	3.36

**Table 2:** Levels of orthogonal experimental factors.

Level	WR	C (kg/m^3^)	FA (%)	CA (%)
1	0.40	330	0	0
2	0.42	340	25	25
3	0.44	350	50	50
4	0.46	360	75	75
5	—	—	100	100

**Table 3:** Mix designs of the 20 orthogonal groups (kg/m^3^).

Specimen	WR	Cement	Fly Ash	Water	Superplasticizer	Fine MGPA Level	MGPA Fine	Sand	Coarse MGPA Level	MGPA Coarse	Gravel
S1	0.40	360	59.4	144.0	4.2	0	0	203.9	0	0	444.0
S2	0.40	360	59.4	144.0	4.2	25	27	152.9	25	48	333.1
S3	0.40	360	59.4	144.0	4.2	50	54	102.0	50	95	222.0
S4	0.40	360	59.4	144.0	4.2	75	82	51.0	75	143	111.0
S5	0.40	360	59.4	144.0	4.2	100	109	0	100	190	0
S6	0.42	350	63.0	147.0	4.2	0	0	185.9	0	0	405.0
S7	0.42	350	63.0	147.0	4.2	50	50	92.9	75	130	101.2
S8	0.42	350	63.0	147.0	4.2	25	25	139.4	100	173	0
S9	0.42	350	63.0	147.0	4.2	100	99	0	25	43	303.6
S10	0.42	350	63.0	147.0	4.2	75	74	46.5	50	87	202.4
S11	0.44	340	66.6	149.6	4.2	0	0	167.4	0	0	365.0
S12	0.44	340	66.6	149.6	4.2	75	67	41.9	100	156	0
S13	0.44	340	66.6	149.6	4.2	100	89	0	50	117	91.2
S14	0.44	340	66.6	149.6	4.2	50	45	83.7	50	78	182.3
S15	0.44	340	66.6	149.6	4.2	25	22	125.6	25	39	273.5
S16	0.46	330	70.2	151.8	4.2	0	0	148.6	0	0	324.0
S17	0.46	330	70.2	151.8	4.2	100	79	0	50	69	161.9
S18	0.46	330	70.2	151.8	4.2	75	59	37.2	25	35	242.8
S19	0.46	330	70.2	151.8	4.2	50	40	74.3	100	139	0
S20	0.46	330	70.2	151.8	4.2	25	20	111.5	75	104	80.9

Note: S19 contains 74.3 kg/m^3^ of sand, so the fine MGPA replacement is partial rather than 100%. The only mixture with 100% replacement of both fine and coarse MGPA is S5.

**Table 4:** Range analysis for the dry density of the MGPA concrete samples.

Parameter	WR	C (kg/m^3^)	FA (%)	CA (%)
K_1_	9297	8195	7926	7926
K_2_	9090	8757	6988	7285
K_3_	8757	9090	6862	7047
K_4_	8195	9297	6837	6766
K_5_	—	—	6726	6315
$k_{1}$	1859.4	1639.0	1981.5	1981.5
$k_{2}$	1818.0	1751.4	1747.0	1821.3
$k_{3}$	1751.4	1818.0	1715.5	1761.8
$k_{4}$	1639.0	1859.4	1709.3	1691.5
$k_{5}$	—	—	1681.5	1578.8
R	220.4	220.4	300	402.7

Note: Due to the complete confounding between WR and C, the identical R values are mathematically inevitable and do not imply equal actual effects.

**Table 5:** Range analysis for the compressive strength of the MGPA concrete samples (MPa).

Factor	WR	C	FA	CA
7-d				
K_1_	145.4	106.2	121.0	121.0
K_2_	152.2	112.6	101.1	105.3
K_3_	112.6	152.2	104.8	106.2
K_4_	106.2	145.4	93.9	98.1
K_5_	–	–	95.6	85.8
$k_{1}$	29.08	21.24	30.25	30.25
$k_{2}$	30.44	22.52	25.28	26.33
$k_{3}$	22.52	30.44	26.20	26.55
$k_{4}$	21.24	29.08	23.48	24.53
$k_{5}$	–	–	23.90	21.45
R	9.20	9.20	6.78	8.80
28-d				
K_1_	182.0	137.8	153.8	153.8
K_2_	192.7	114.5	128.7	133.9
K_3_	114.5	192.7	133.4	135.1
K_4_	137.8	182.0	119.3	124.9
K_5_	–	–	121.8	109.3
$k_{1}$	36.40	27.56	38.45	38.45
$k_{2}$	38.54	28.90	32.16	33.48
$k_{3}$	28.90	38.54	33.35	33.78
$k_{4}$	27.56	36.40	29.83	31.23
$k_{5}$	–	–	30.45	27.33
R	10.98	10.98	8.63	11.13

Note: Due to the complete confounding between WR and C, the identical R values are mathematically inevitable and do not imply equal actual effects.

**Table 6:** Mix proportions of ceramsite concrete.

Ceramsite Replacement (%)	Ceramsite (kg/m^3^)	Sand (kg/m^3^)	Cement (kg/m^3^)	Fly Ash (kg/m^3^)	Water (kg/m^3^)	Superplasticizer (kg/m^3^)
15	259.7	935.4	400	50	180	4.76
20	302.5	808	400	50	180	4.76
25	345.3	680.5	400	50	180	4.76

**Table 7:** Dry density and compressive strength of the MGPA concrete samples.

Specimen	Density (kg/m^3^)	Compressive Strength (MPa)	
		7 d	28 d
S1	2089	36.5	45.7
S2	1938	31.3	39.2
S3	1872	31.1	38.9
S4	1767	26.0	32.5
S5	1631	20.5	25.7
S6	2041	34.9	44.2
S7	1781	28.7	36.4
S8	1647	24.9	31.5
S9	1833	32.1	40.6
S10	1788	31.6	40.0
S11	1938	25.5	32.7
S12	1592	18.2	23.3
S13	1639	22.3	28.6
S14	1764	22.8	29.3
S15	1824	23.8	30.6
S16	1858	24.1	31.2
S17	1623	20.7	26.9
S18	1690	18.1	23.5
S19	1445	22.2	28.8
S20	1579	21.1	27.4

Note: The range analysis in this study is based on the mean values of three replicate specimens. The overall trends observed across the 20 orthogonal mixtures were consistent, providing reliable directional guidance for mix proportion optimization.

**Table 8:** Comparison of the physical and mechanical properties of ceramsite and MGPA.

Aggregate	Bulk Density (kg/m^3^)	Apparent Density (kg/m^3^)	Voidage (%)	Water Absorption (%)	Cylinder Strength (MPa)
Ceramsite	678	1026	33.9	13.4	3.8
MGPA	312	668	45.8	18.1	3.36

**Table 9:** Comparison of the optimum mixtures and properties.

Concrete Type	Optimum Mix	Dry Density (kg/m^3^)	28-d Strength (MPa)	Specific Strength (kN·m/kg)	Slump (mm)
Ceramsite	20% ceramsite, WR = 0.45	1710	20.5	12.0	168
MGPA	WR = 0.42, C = 350, FA = 50%, CA = 50% (S10)	1788	40.0	22.4	–

## Data Availability

The original contributions presented in this study are included in the article. Further inquiries can be directed to the corresponding author.
